# Transparent, solvent‐free, and pressure‐tolerant antifouling coatings via molecular nanocomposite engineering

**DOI:** 10.1002/smo2.70068

**Published:** 2026-07-21

**Authors:** Jieran Li, Xiubin Xu, Yueyan Liang, Hongchun Mu, Yang Xue, Jianwei Liu, Daijiang Cai, Yue Lan, Jiahui Tang, Sizhe Wang, Zhenxuan Wei, Xu Wu

**Affiliations:** ^1^ School of Chemistry and Chemical Engineering Guangzhou University Guangzhou China

**Keywords:** anti‐fingerprint, anti‐fouling, dynamic liquid‐repellency, eco‐friendly, protective coatings

## Abstract

Traditional solvent‐based antifouling coatings face critical limitations including application onto miscible organic substrates and volatile organic compound (VOCs) pollution. Herein, this work presented a solvent‐free, transparent antifouling coating through room temperature vulcanization, based on a strategy of molecularly engineered nanocomposite networks. This strategy utilizes hydroxy‐terminated polydimethylsiloxane (HO‐PDMS‐OH) as functional segments to enable dynamic liquid repellency, isocyanatopropyltriethoxysilane (IPTS) and 3‐(2‐aminoethylamino) propylmethyldimethoxysilane (AEAPS) as dual cross‐linkers for rapid curing and robust network, and hydrophobic nano‐SiO2 as reinforcing fillers for anti‐fouling and mechanical robustness. The coating exhibited low VOC release through solvent‐free processing, high visible‐light transmittance (91% at 500 nm), and broad‐spectrum repellency with excellent durability against abrasion/chemical exposure/long‐term oil immersion. This unique coating strategy makes it possible for the ready application of antifouling technology onto heat‐sensitive, solvent‐sensitive, and flexible substrates such as flexible display devices. This work establishes an eco‐friendly paradigm for multifunctional protective coating through molecular nanocomposite engineering.

## Graphical abstract

This work developed a solvent‐free, room temperature–curable transparent coating. Featuring an ultrasmall nanocomposite network, it achieves robust liquid‐repellent and anti‐fingerprint properties with eco‐friendly and scalable potential for multifunctional protection.

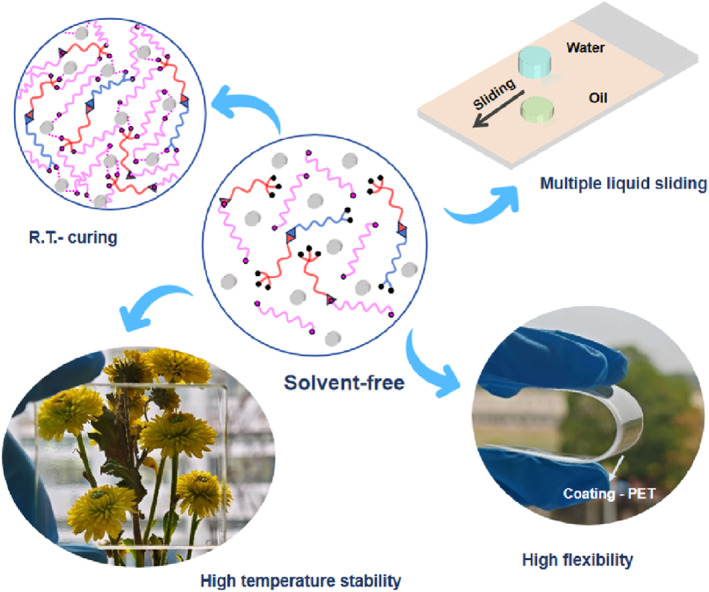

## 1 INTRODUCTION

The rapid advancement of industrialization and urbanization has driven an exponential growth in the demand for high‐performance antifouling coatings across diverse sectors, including biomedical applications,[^1^, ^2^, ^3^] oil‐water separation,[^4^, ^5^] marine applications,[^6^, ^7^, ^8^, ^9^] and electronic devices.[^10^, ^11^, ^12^, ^13^, ^14^, ^15^, ^16^] However, conventional antifouling coatings suffer from critical limitations that severely constrain their widespread application.[^17^, ^18^, ^19^, ^20^] Firstly, their preparation typically requires the use of organic solvents (e.g., toluene and acetone) as dispersion media but results in result in substantial emissions of volatile organic compounds (VOCs) during both manufacturing and application process.[^10^, ^21^, ^22^] This not only leads to environmental pollution but also poses significant health risks.[^23^, ^24^, ^25^] Secondly, the curing of these coatings typically necessitates elevated temperatures or ultraviolet (UV) radiation, thereby restricting their applicability on flexible, thermally unstable substrates and complex operational environments.[^26^, ^27^] Thirdly, the anti‐fouling efficacy of conventional coatings, particularly those inspired by the lotus effect, which rely on trapped air layers and specific surface topography, is prone to deterioration during practical applications.^[28]^ This vulnerability arises from their susceptibility to failure under pressure, as liquids can infiltrate the texture and displace the trapped air, leading to compromised performance under conditions such as mechanical abrasion or prolonged underwater immersion.[^29^, ^30^, ^31^]

In response to these challenges, room‐temperature (RT) curing technology has attracted great attention due to its potential to reduce energy consumption and expand application scenarios, particularly for heat‐sensitive substrates like plastics and textiles.[^32^, ^33^] Nevertheless, the implementation of RT curing technology faces two principal hurdles: (i) sluggish reaction kinetics at ambient temperatures, which complicate the balancing of curing dynamics and crosslinking efficiency, often leading to incomplete curing[^34^, ^35^, ^36^]; and (ii) issues related to prepolymer stability and phase separation between functional components, such as antifouling agents, and the resin matrix, which can compromise the coating's overall performance.[^37^, ^38^] Consequently, the development of a superior antifouling coating that combines the features of RT curing, environmental friendliness, minimal VOC emissions, and pressure tolerance holds substantial scientific and engineering significance.[^39^, ^40^, ^41^, ^42^]

Herein, we reported a RT curable silicone coating via molecular nanocomposite engineering that transcends conventional trade‐offs. As shown in Supporting Information S1: Figures S1 and S2, low‐molecular‐weight hydroxy‐terminated polydimethylsiloxane (HO‐PDMS‐OH) was employed as both the foundational polymer matrix and a key antifouling component to ensure excellent chain flexibility for dynamic liquid repellency. 3‐(2‐aminoethylamino)propylmethyldimethoxysilane (AEAPS) and isocyanatopropyltriethoxysilane (IPTS) as dual cross‐linkers and hydrophobic nano‐SiO_2_ as a reinforcing filler to achieve mechanical resilience and chemical inertness, as well as good transparency. Under ambient temperature and catalyzed by ditin butyl dilaurate (T12), a stable silicone prepolymer was obtained through the nucleophilic addition reaction between isocyanate groups and amino/silanol groups. This prepolymer subsequently underwent solvent‐free RT curing to form coating on diverse substrates through a moisture‐curing mechanism. The resultant coating exhibited excellent liquid repellency, outstanding antifouling efficacy, good mechanical durability, and chemical stability. Its RT curing characteristic successfully endowed the coating with broad substrate applicability (including heat‐sensitive substrates) and potential for large‐scale application. Simultaneously, the coating demonstrates high transparency in the visible light region, achieving a transmittance of 91% at 500 nm. This work not only establishes generalizable framework for integrating dynamic interfacial engineering with nanocomposite materials science but also addresses a critical gap in multifunctional coating design for the development of comprehensive high‐performance antifouling coatings.

## 2 RESULTS AND DISCUSSION

**FIGURE 1:**
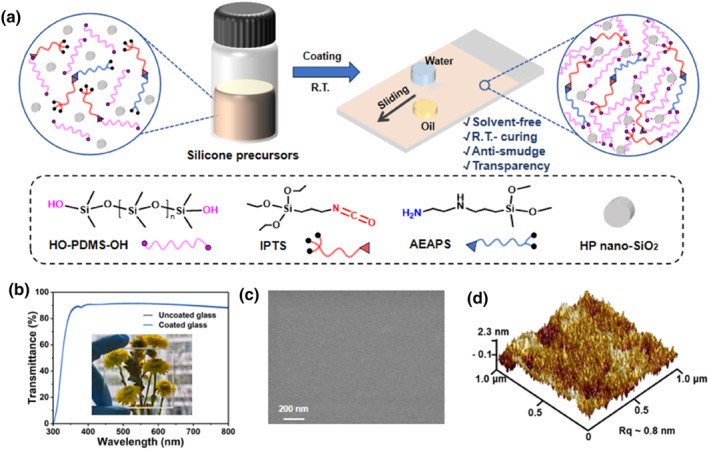
(a) Schematic diagram of coating preparation. (b) Transmittance of coating. (c) TEM image. (d) atomic force microscopy image.

A one‐component room temperature (RT)–curable silicone solution was first constructed, involving the solvent‐free, sealed mixing of a monomer, cross‐linkers, a catalyst, and a reinforcing filler at room temperature to form a silicone prepolymer (Figure 1a). During application, the prepolymer underwent further hydrolysis and de‐alcohol condensation upon exposure to atmospheric moisture, ultimately curing to form an antifouling coating. Fourier transform infrared (FTIR) spectroscopy analysis (Supporting Information S1: Figure S3) confirmed the reaction completion that, compared to those of IPTS and AEAPS, the coating exhibited no characteristic peaks for N=C=O (2275 cm^−1^), ‐NH_2_ (3340 cm^−1^), Si‐OH (3435 cm^−1^), and a characteristic peak at 1256 cm^−1^, corresponding to the overlapping vibrations of the urea C=O stretch and methyl C‐H bend. These results confirmed that the ‐NH_2_ and Si‐OH groups reacted completely with the excess ‐NCO groups. After curing, the coating exhibited a high optical transparency that the transmittance curve of the coating closely matches that of the bare glass substrate, achieving a high visible‐light transmittance of 91% at 500 nm, as shown in Figure 1b. Such good transparency was attributed to the dense and homogeneous structure of the coating that the hydrophobic nano‐silica as the reinforcing filler maintained the inherent transparency due to its nanoscale dimensions, thereby imposing no adverse effect on the coating's optical clarity.[^39^, ^43^, ^44^, ^45^] Furthermore, its ultra‐small size ensured that it did not alter the coating's surface morphology. The scanning electron microscopy (SEM) images of the coating surface confirmed the hypothesis that the structure of the coating was a uniform and densely ultrasmall nanoparticle‐packed pattern (Figure 1c), which was also confirmed by the results of the elemental distribution mapping that both the surface (Supporting Information S1: Figure S4) and the cross‐section (Supporting Information S1: Figure S5) revealed a uniform distribution. The primary constituent elements display consistent distribution across micro‐regions within the coating's cross‐section and depth (Supporting Information S1: Table S1), with no significant elemental enrichment observed. This confirms the structural homogeneity of the coating material at the microscopic scale. Such uniform elemental distribution established a robust foundation for the functional consistency and stability of the coating across macroscopic areas.[^43^, ^46^] The atomic force microscopy (AFM) result displayed that the coating surface possessed an extremely low root mean square (RMS) roughness (Rq ≈ 0.8 nm), as shown in Figure 1d. This indicates a high degree of smoothness and uniformity at the nanoscale, consistent with the SEM analysis and providing additional validation of the coating's exceptionally flat and smooth surface characteristics. The X‐ray photoelectron spectroscopy (XPS) analysis (Supporting Information S1: Figure S6) indicates no significant difference in elemental composition and relative concentrations between the two interfaces of the coating (Supporting Information S1: Table S2). This finding is consistent with the EDS‐SEM results, providing additional validation of the coating material's structural homogeneity. Notably, silicon (Si), which exhibits weaker binding affinity with liquids, constitutes a significantly high proportion (>30%) of the coating composition. In contrast, nitrogen (N), which typically exhibits stronger binding affinity with many materials, is present at a relatively low concentration (<2%) (Supporting Information S1: Figure S7). This specific elemental composition characteristic contributes to a low surface energy for the coating surface, thereby endowing the coating with excellent antifouling performance.[^37^, ^47^, ^48^, ^49^]

**FIGURE 2:**
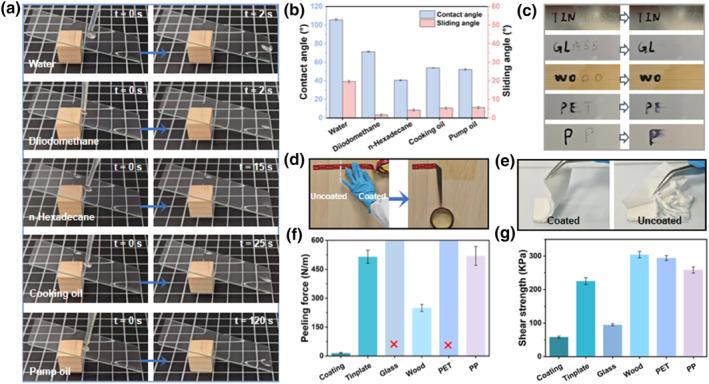
(a) Photographs of various liquids sliding off the coated glasses without leaving any residue. (b) Contact angles and sliding angles of various liquids. (c) The coatings applied on different substrates all have a hydrophobic effect. Comparison of the adhesion performance of the surface of the substrate with or without coating to 3M strong adhesive (d) and double‐sided sponge adhesive (e). (f) The peel force test results of 3M super glue on different substrate surfaces. (g) The shear strength of 3M sponge double‐sided adhesive on different substrate surfaces.

Thus, the antifouling capability of the coating against liquids was evaluated, as shown in Figure 2a and Movie S1. Various liquids, including water (surface tension: 72.0 mN/m at 20°C),^[50]^ diiodomethane (50.0 mN/m),^[51]^ and n‐hexadecane (27.5 mN/m),^[52]^ as well as oily liquids such as edible oil and pump oil, could completely roll off the tilted coating surface without any residue, indicating the excellent anti‐fouling performance to a wide range of liquids. To further quantitatively evaluate the anti‐fouling performance of the coating, the contact angles (CAs) and sliding angles (SAs) of the liquids on the coating surface were determined, as in Figure 2b. Water exhibited a high CA of ∼105° on the coating surface, while the diiodomethane and n‐hexadecane exhibited a lower CAs than the water due to their lower surface tensions. Both edible oil and pump oil displayed low CAs (<60°) and SAs (<10°). Specifically, the SAs of non‐aqueous liquids (≤6°) below that of water (∼20°) may be attributed to the formation of a highly flexible, liquid‐like surface layer of silicone oil at the flexible coating with a low glass transition temperature (Tg) (Supporting Information S1: Figure S8), thus facilitating the weaker interface‐interacted liquid sliding. To further validate the liquid‐repellent performance originating from its liquid‐like surface, the coating was immersed in petroleum ether to extract any free silicone oil, and its surface property was characterized by the water contact angles (WCA), water sliding angles (WSA), hexadecane CAs (OCA), and hexadecane SAs (OSA). As shown in Supporting Information S1: Figure S9, all angles showed negligible variation throughout the 4‐week immersion test, indicating the long‐term stability of the coating's surface properties and that its anti‐fouling performance was attributed to the high mobility of the grafted PDMS chains. Moreover, this anti‐fouling coating could be applied on various substrates, such as tinplates, glass, wood, Polyethylene terephthalate (PET), and polypropylene (PP), as shown in Figure 2c and Movie S2.

Beyond the low adhesion exhibited toward liquids, the coating also demonstrated significant resistance to adhesion by sticky solid contaminants. As evidenced in Figure 2d–e and Movie S3, while two kinds of the commercial tapes adhered strongly to the bare substrate, they showed negligible adhesion to the coated surface. Peeling force testing revealed that the tape exhibited a remarkably low peel force of only ∼15.15 N/m on the coated surface, significantly lower than that measured on other bare substrates (Figure 2f). Notably, the peeling forces of the tape on both the bare glass and PET were exceedingly high, leading to severe stretching deformation of the tape during testing (Supporting Information S1: Figure S10). Furthermore, the shear strength of the double‐sided foam tape on the coated surface was approximately 58.17 kPa, significantly lower than that on bare tinplate (∼225.26 kPa), glass (∼94.78 kPa), wood (∼304.21 kPa), PET (∼293.97 kPa), and PP (∼258.15 kPa), as shown in Figure 2g. These results confirmed the excellent low adhesion properties of the coating.

**FIGURE 3:**
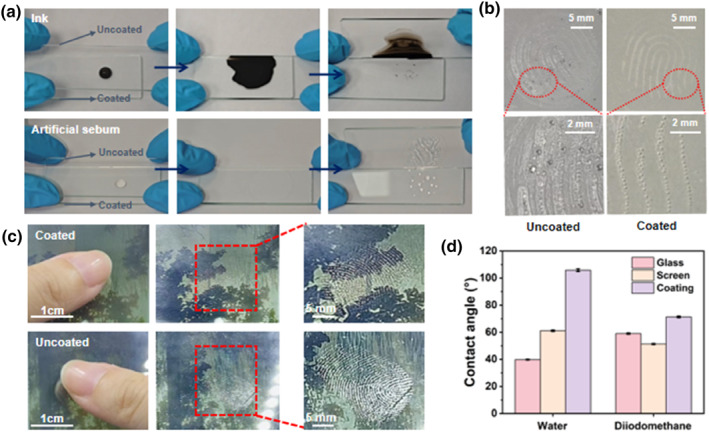
(a) The coating maintained its liquid repellency and anti‐smudge performance when it was subjected to local pressure resulting from two pieces of squeezed glasses, tested with water‐based ink. (b) The spreading behavior of artificial sebum droplets with or without coating. (c) The application of coatings on the screens of smart phones. (d) The contact angles of water and n‐hexadecane on the coating, screen and glass surfaces.

It is well‐known that Lotus‐inspired superhydrophobic or superoleophobic surfaces are susceptible to failure under pressurized liquids, as the liquid tends to infiltrate the texture and displace the trapped air.^[53]^ Distinct from these pressure‐sensitive rough surfaces, this coating could effectively maintain its antifouling performance even under pressure. As shown in Figure 3a and Movie S4, droplets of hydrophilic ink and artificial sebum were placed on the coated glass and then pressured by the other bare glass with obvious wetting phenomena. However, after removing the top bare glass, these droplets were spread and strongly adhered to the uncoated glass, while rapidly contracted and changed into smaller, discrete droplets on the coated surface. These results imply that this coating could be used to anti‐fingerprint to reduce the contaminated area and protect personal privacy. As shown in Figure 3b, artificial sebum exhibited significant wetting behavior on the uncoated glass, forming a continuous liquid film with a clear fingerprint pattern. Conversely, the sebum spontaneously retracted into isolated small spherical droplets on the coated surface. These distinct wetting behavior confirmed that the coating could effectively prevent the fingerprint residue. Figure 3c demonstrated the successful potential application of the coating used in smartphone screen that the fingerprint of a finger with artificial sebum obviously obscured the underlying screen content with a continuous liquid film for bare glass, while exhibited liquid‐repellent behavior and the underlying pattern remained could be identified for the coated glass. This result was also confirmed by the fluorescent images (Supporting Information S1: Figure S11). Figure 3d demonstrated that both water and n‐hexadecane exhibited significantly higher CAs on the coated surface compared to bare glass and the screen substrate itself. This difference is attributed to the lowest surface energy disparities of the coating then the glass and screen (Supporting Information S1: Figure S12). These results confirmed that the coating possesses excellent low adhesion towards complex mixtures like artificial sebum and significant potential for application demonstrating significant potential compared to other coatings (Supporting Information S1: Figure S13) for anti‐fingerprint functional materials in smartphone screens or medical touch screen devices.

**FIGURE 4:**
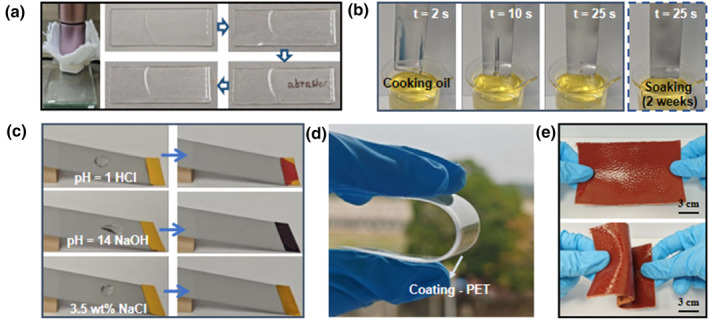
(a) Before and after the cyclic friction test, photos of the graffiti. (b) Test of anti‐sticking property of coating when immersed in cooking oil. (c) The situation of the liquid sliding off the coating surface after being treated with a strong acid and strong base salt solution. (d) The coating can be applied onto polyethylene terephthalate and is flexible. (e) The coating can be applied to the leather and can be bent to any degree and position for folding and twisting.

For practical applications, long‐term stability of the coating's performance under various harsh conditions are vital;^[54]^ for example, smartphone screens are frequently subjected to contact with soft materials like fabric and skin, which require durable resistance to such low‐load physical abrasion.^[55]^ Therefore, cyclic abrasion tests were conducted on the coating, as illustrated in Figure 4a. After several cycles of abrasion, the oil‐based ink markings on the surface could still be readily cleaned, indicating the capability to withstand typical daily wear and tear. It may be attributed to the coating's uniform and dense three‐dimensional network structure, where all micro‐regions exhibit homogeneous low surface energy characteristics. Consequently, the coating maintained good functional stability after physical abrasion. However, the anti‐fouling efficacy of such soft coating may be lost when subjected to high‐load wear scenarios, due to its inherent mechanical softness (pencil hardness of H). To further assess the long‐term stability of the anti‐oil‐fouling performance under everyday conditions, the coating was immersed in cooking oil for 14 days (at 25°C), and the results demonstrated that the coating retained its anti‐fouling efficacy without significant degradation (Figure 4b and Movie S5), indicating the excellent oil‐repellent capability and functional durability. Additionally, considering potential exposure to aggressive chemical environments during service life, the coating was tested against strong acids (1 M HCl, pH = 1), strong bases (1 M NaOH, pH = 14), and salt solutions (3.5 wt% NaCl) and the excellent anti‐fouling behavior was still maintained for the coated surface (Figure 4c). More rigorous immersion tests further confirmed the coating's robustness: the coated tinplate maintained its metallic luster without visible signs of corrosion after 24 h in strong acid, 10 h in strong base, and 20 days in salt solution; all of the above were under room temperature conditions of 25°C. Furthermore, the coating's anti‐graffiti functionality remained intact and the oil‐based ink markings could still be easily wiped clean (Supporting Information S1: Figure S14). These results confirmed the exceptional stability and retained functionality of the coating even in extreme chemical environments.

Furthermore, as shown in Figure 4d, the coating possessed excellent flexibility that it remained intact without cracking or delamination even after the PET was bent into a U‐shape. Similarly, the coating showed no signs of physical damage when applied to leather, which was subjected to repeated folding and twisting at various angles and positions (Figure 4E). Coupled with its strong adhesion to diverse substrates (Supporting Information S1: Figure S15) and compatibility with industrial‐scale coating processes (Supporting Information S1: Figure S16), this coating demonstrates significant practical potential for applications in industrial protection, construction, and related fields based on the ultrasmall functional building blocks strategy. Thus, the coating could effectively meet the comprehensive performance requirements and large‐scale application demands for real‐world industrial applications.

## 3 CONCLUSION

This work presented a solvent‐free, transparent antifouling coating fabricated via room temperature vulcanization of a tailored silicone system. The cross‐linked network, formed through nucleophilic addition and moisture‐triggered condensation, yields a dense, homogeneous structure with exceptionally low surface energy and a flexible liquid‐like interface. This design confers outstanding broad‐spectrum repellency, remarkably low adhesion to sticky solids, and pressure‐tolerant anti‐fouling performance, effectively preventing fingerprint contamination. The coating simultaneously achieves high optical clarity (91% transmittance @500 nm), nanoscale smoothness (Rq ≈ 0.8 nm), and robust durability against abrasion, long‐term oil immersion, and harsh chemicals (acids, bases, and salt), while protecting substrates from corrosion. Its excellent flexibility on diverse materials (PET, leather) and compatibility with industrial‐scale processes enable versatile applications. Combining eco‐friendly fabrication (VOC‐free, RT‐curing) with multifunctional performance, this coating offers significant potential for demanding uses in anti‐fingerprint displays, medical devices, industrial protection, and architectural materials.

## 4 EXPERIMENTAL SECTION

### 4.1 Materials

Hydroxy‐terminated polydimethylsiloxane (PDMS) was purchased from Wacker Chemie AG. Isocyanatopropyltriethoxysilane (IPTS), 3‐(2‐aminoethylamino) propylmethyldimethoxysilane (AEAPS), ditin butyl dilaurate (T12), silicon dioxide (SiO_2_), diiodomethane, n‐hexane, and hexadecane were purchased from Macklin. HCl, NaCl, NaOH was purchased from Tianjin Damao. Cooking oil and pump oil were purchased from local stores.

### 4.2 Preparation of the transparent silicone prepolymer

A mixture of 7 g of low‐molecular‐weight hydroxyl‐terminated silicone oil and 1.8 g of IPTS was stirred in a sealed container for 2 min. Subsequently, 0.9 g of AEAPS was added dropwise. After completion of the addition, stirring was maintained until the temperature decreased to ∼40°C (about 5–10 min). Thereafter, 0.1 g of T12 was added, and stirring continued for 1 h. Finally, 0.1 g of SiO_2_ was added, and the mixture was stirred thoroughly for 24 h to yield a transparent silicone prepolymer. The entire procedure was conducted at room temperature under magnetic stirring with a stirring rate of 450 rpm.

### 4.3 Preparation of the coating

The transparent silicone prepolymer was coated onto the substrate and then the substrate was tilted at a 45‐degree angle and maintained under ambient conditions for 3 days, yielding the coating.

### 4.4 Surface chemical characterization of the coating

The molecular structure of the coating surface was analyzed using Attenuated Total Reflection Fourier‐Transform Infrared Spectroscopy (ATR‐FTIR). For comparative analysis, conventional Fourier‐Transform Infrared Spectroscopy (FTIR) was employed to characterize the individual components. All measurements were performed across a wavenumber range of 400 to 4000 cm^−1^. The Fourier transform infrared spectrometer used was the Bruker TENSOR II + Hyperion 2000.

### 4.5 Coating morphology characterization

The surface and cross‐sectional microstructures of the coating were characterized by scanning electron microscopy (SEM). Surface topography and roughness were further quantified using AFM. SEM images of coatings were obtained by a Zeiss Sigma 300 scanning electron microscope at 5 KV. Coatings were sputtered with gold via Quorum SC7620 before SEM observations. AFM images of coatings were obtained using a Bruker Dimension Iconscanning probe microscope.

### 4.6 Elemental distribution and composition analysis of the coating

Elemental mapping analysis of the coating surface and cross‐section was performed via energy‐dispersive X‐ray spectroscopy (EDS) coupled with scanning electron microscopy (SEM) to determine local elemental distribution. Additionally, XPS survey scans were conducted on the top and bottom surfaces to determine their elemental composition and perform quantitative analysis. The EDS analysis was performed using a Zeiss Sigma 300 scanning electron microscope equipped with an energy‐dispersive X‐ray spectroscopy system. X‐ray Photoelectron Spectroscopy (XPS, K‐Alpha, Thermo Fisher Scientific) equipped with monochromatized Al Kα radiation (hυ = 1486.7 eV) was employed to analyze the elemental composition of the coating surface.

### 4.7 Determination of coating glass transition temperature

The glass transition temperature (Tg) of the coating was determined by differential scanning calorimetry (DSC). Measurements were performed employing heating‐cooling cycles between −100°C and 100°C at a rate of 10°C/min under a nitrogen atmosphere. Tests were conducted using the NetZsch STA449F5.

### 4.8 Surface contact angle measurement of the coating

Static CAs and SAs (SA) of various liquids on the coating surface were determined using a dynamic CA analyzer (JC2000A from Dongguan Shengding Precision Instruments Co., Ltd.). Test liquids included water, diiodomethane, n‐hexadecane, edible oil, and pump oil. Measurements were performed with deposition volumes of 5 μL for CAs and 20 μL for SAs, delivered via a microsyringe.

### 4.9 Tensile measurements

The samples were evaluated using a universal testing machine (HZ‐1007E, Dongguan Lixian Instrument Technology Co., Ltd) at a rate of 50 mm min^−1^. A sample was made by pressing 3m strong adhesive onto the coated substrate surface. A pulling force was applied to the samples until a separation occurred between the coating and the substrate, then the maximum stress and strain values were recorded. Shear strength was determined as the ratio of the maximum stress and the coating area. At least five parallel samples were tested for each group.

### 4.10 The mechanical stability of the coating

The mechanical stability of the coating was assessed by performing mechanical abrasion on its surface with a Model A20‐339 abrasion tester (Suzhou Chuangheng Electronic Instrument Co., Ltd.) using a cotton cloth as the contact material under a 500 g load, followed by evaluation of the anti‐graffiti performance on the abraded surface that exhibited obvious scratches.

### 4.11 Transparency test of the coating

The transparency of the coating was evaluated using a PerkinElmer Lambda 950 UV/Vis spectrophotometer. The transmittance was measured over a wavelength range of 300–800 nm, with air as the baseline. The transmittance of an uncoated glass slide and a coated glass slide was measured, and the transmittance at 500 nm was taken as the visible light transmittance of the sample.

### 4.12 Adhesion test of the coating

At room temperature, a commercially available 3M sponge double‐sided adhesive tape was attached to the surfaces of both uncoated and coated glass substrates, and then peeled off using tweezers.

### 4.13 Anti‐fingerprint test of the coating

The anti‐fingerprint performance of the coating was evaluated as follows. An artificial fingerprint liquid (produced by Dongguan Chuangfeng Automation Co., Ltd.) was dropped onto a tissue paper until it was fully saturated. A finger was then dipped into the artificial fingerprint liquid on the tissue paper and pressed onto the surface of a smartphone with and without the coating using a constant force. The residual fingerprint liquid on the surface was observed.

## CONFLICT OF INTEREST STATEMENT

The authors declare no conflicts of interest.

## ETHICS STATEMENT

No animal or human experiments were involved in this study.

## Supporting information

Supporting Information S1

Movie S1

Movie S2

Movie S3

Movie S4

Movie S5

## Data Availability

The data that support the findings of this study are available from the corresponding author upon reasonable request.
