# Supplementary material for: Transparent, solvent‐free, and pressure‐tolerant antifouling coatings via molecular nanocomposite engineering
Source: Smart Mol. 2026 Jul 21:e70068. Online ahead of print. doi: 10.1002/smo2.70068 (PMC13399332; doi:10.1002/smo2.70068)
Supplement: Supplementary file 1 — Supporting Information S1 [file SMO2-9999-0-s004.docx]

Supplementary Materials for

**Transparent, solvent-free, and pressure-tolerant antifouling coatings via molecular nanocomposite engineering**

Jieran Li^#^, Xiubin Xu^#^, Yueyan Liang, Hongchun Mu, Yang Xue, Jianwei Liu, Daijiang Cai, Yue Lan, Jiahui Tang, Sizhe Wang, Zhenxuan Wei, Xu Wu*

School of Chemistry and Chemical Engineering, Guangzhou University, Guangzhou 510006, China

^*^ Corresponding authors.

^#^ These authors contributed equally to this paper.

E-mail address: xuwu@gzhu.edu.cn (X. Wu)


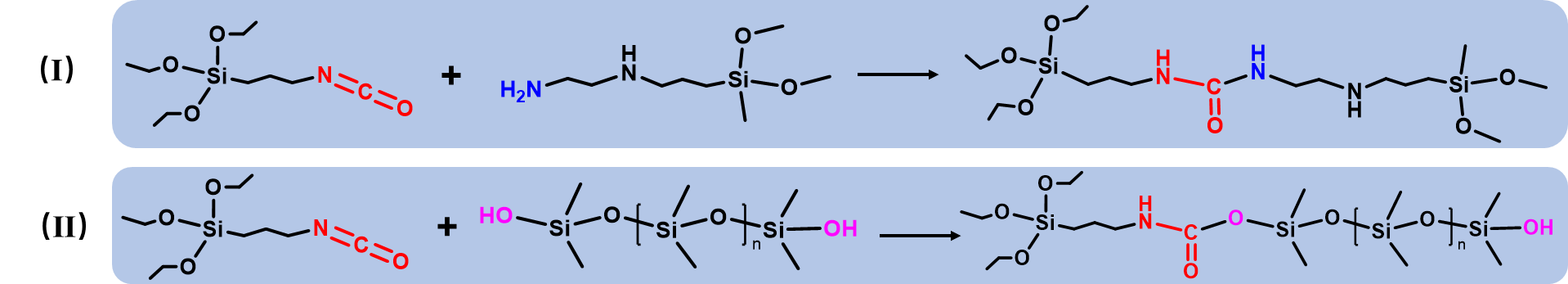


**Figure S1.** Chemical reaction in the synthesis of prepolymers.


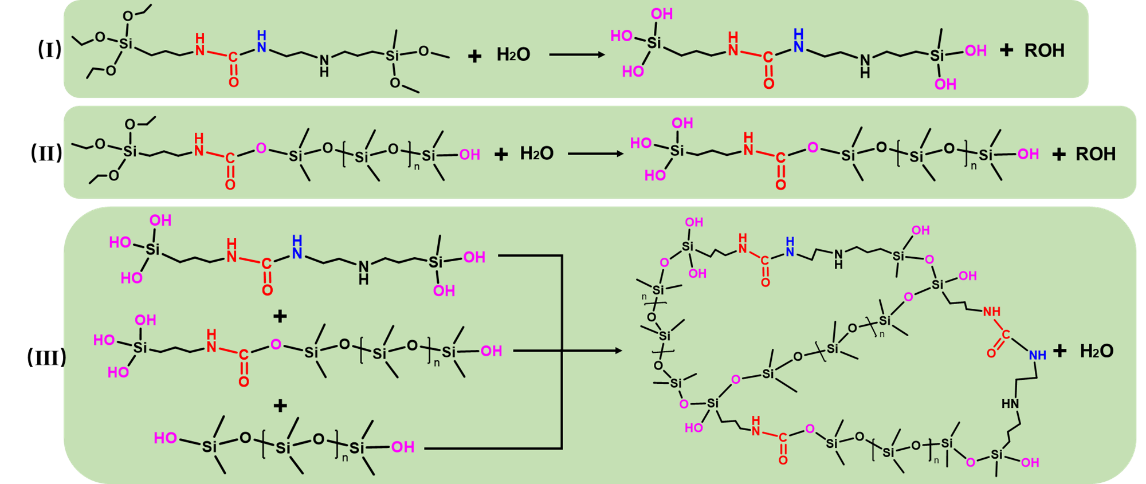
**Figure S2.** Chemical reactions occurring during the crosslinking and curing prcess of the coating.


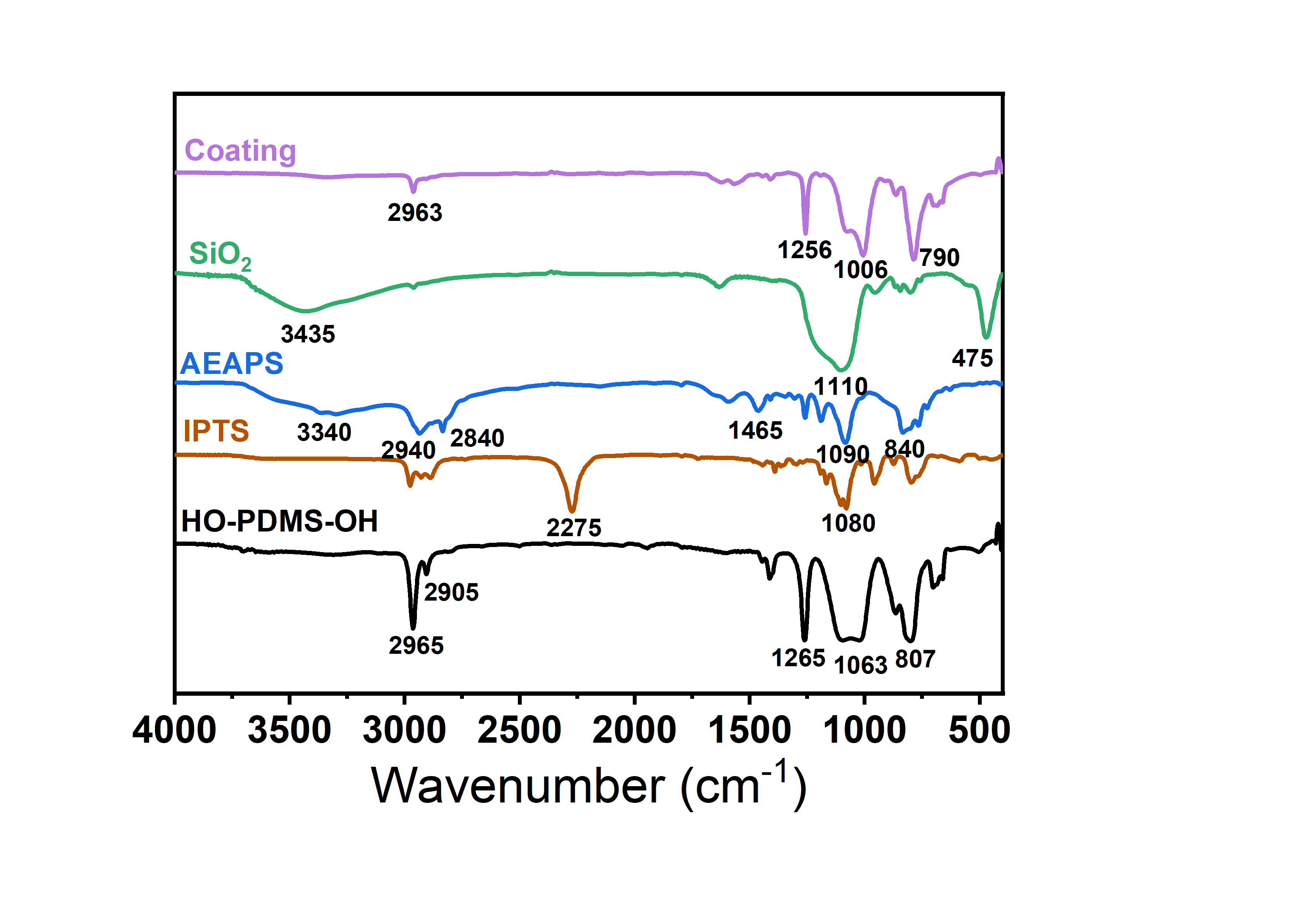
**Figure S3.** Infrared spectra of each component of the raw material and the coating.


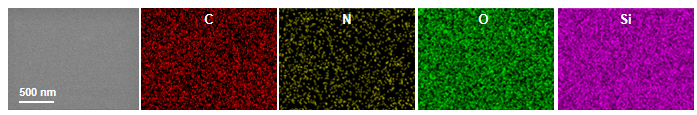


**Figure S4.** Analysis of surface elements of the coating.


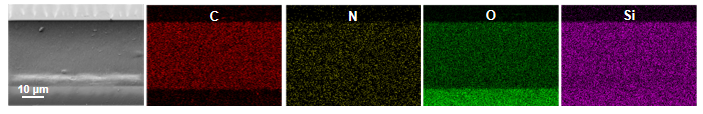


**Figure S5.** Analysis of coating cross-sectional elements.


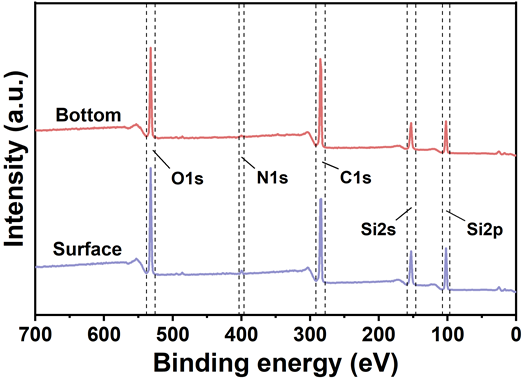


**Figure S6.** Coating surface and bottom surface XPS.


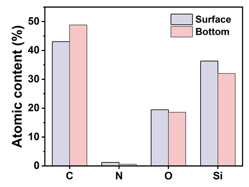


**Figure S7.** Distribution of elements on the bottom surface of the coating layer.


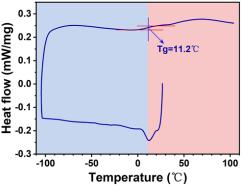


**Figure S8.** DSC curve of the coating.


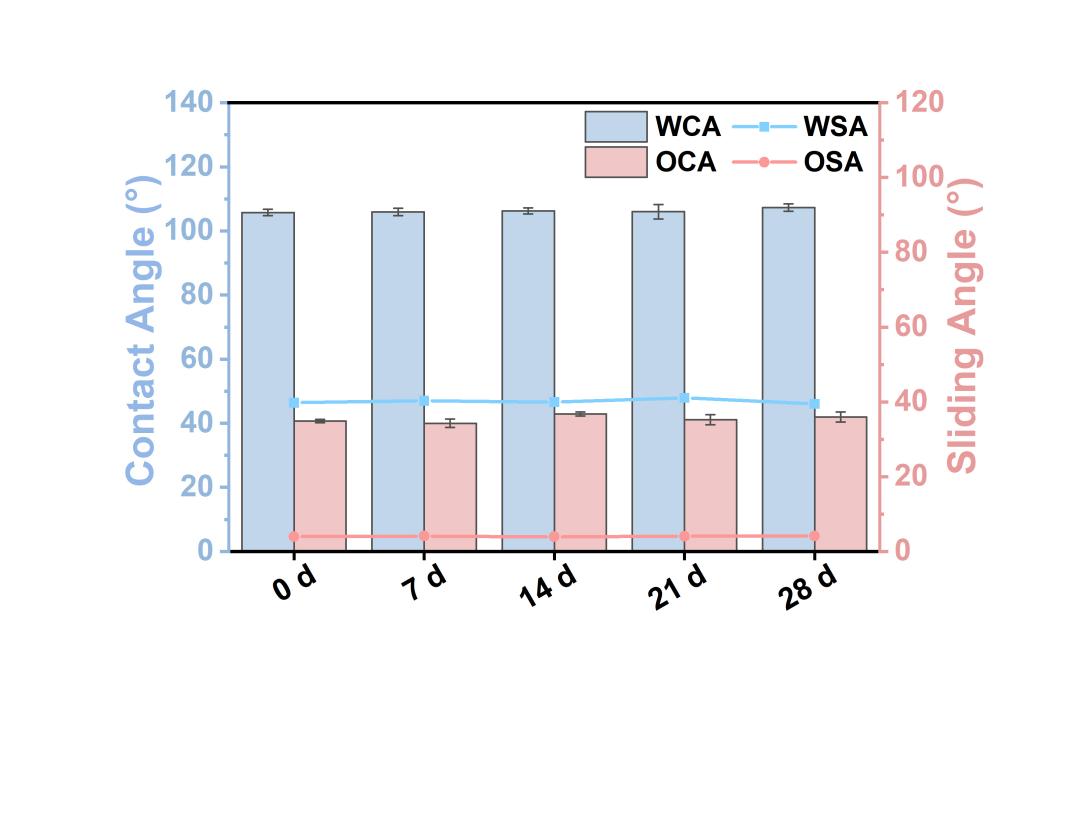


**Figure S9.** The contact angles and sliding angles of water and hexadecane on the coating surface after 28 days of immersion in petroleum ether.


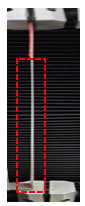


**Figure S10.** The adhesive tape underwent severe stretching deformation during the test, and even when the displacement limit of the testing instrument was reached, it still failed to be completely peeled off.


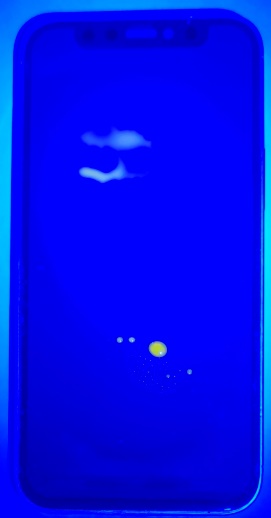

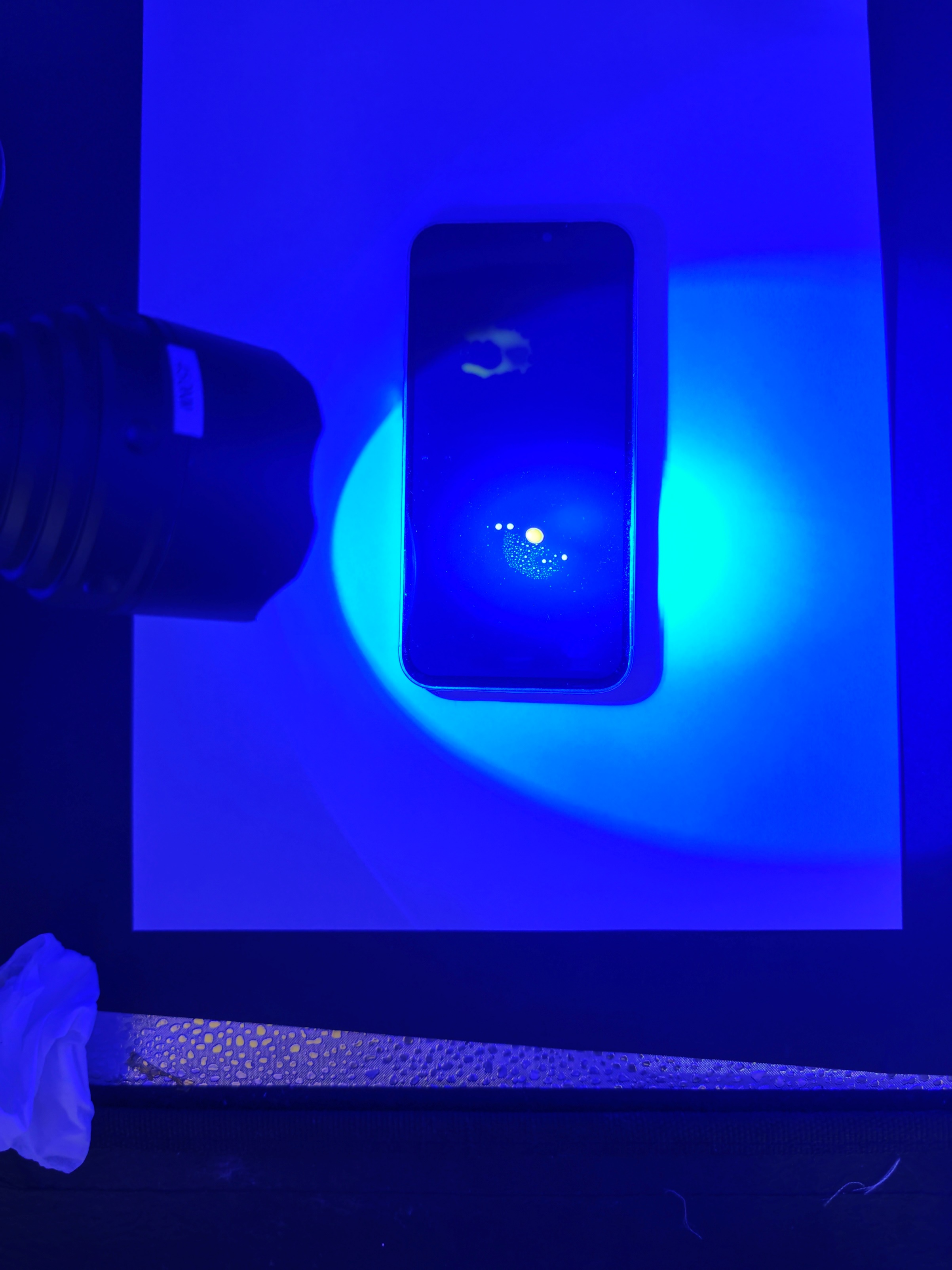

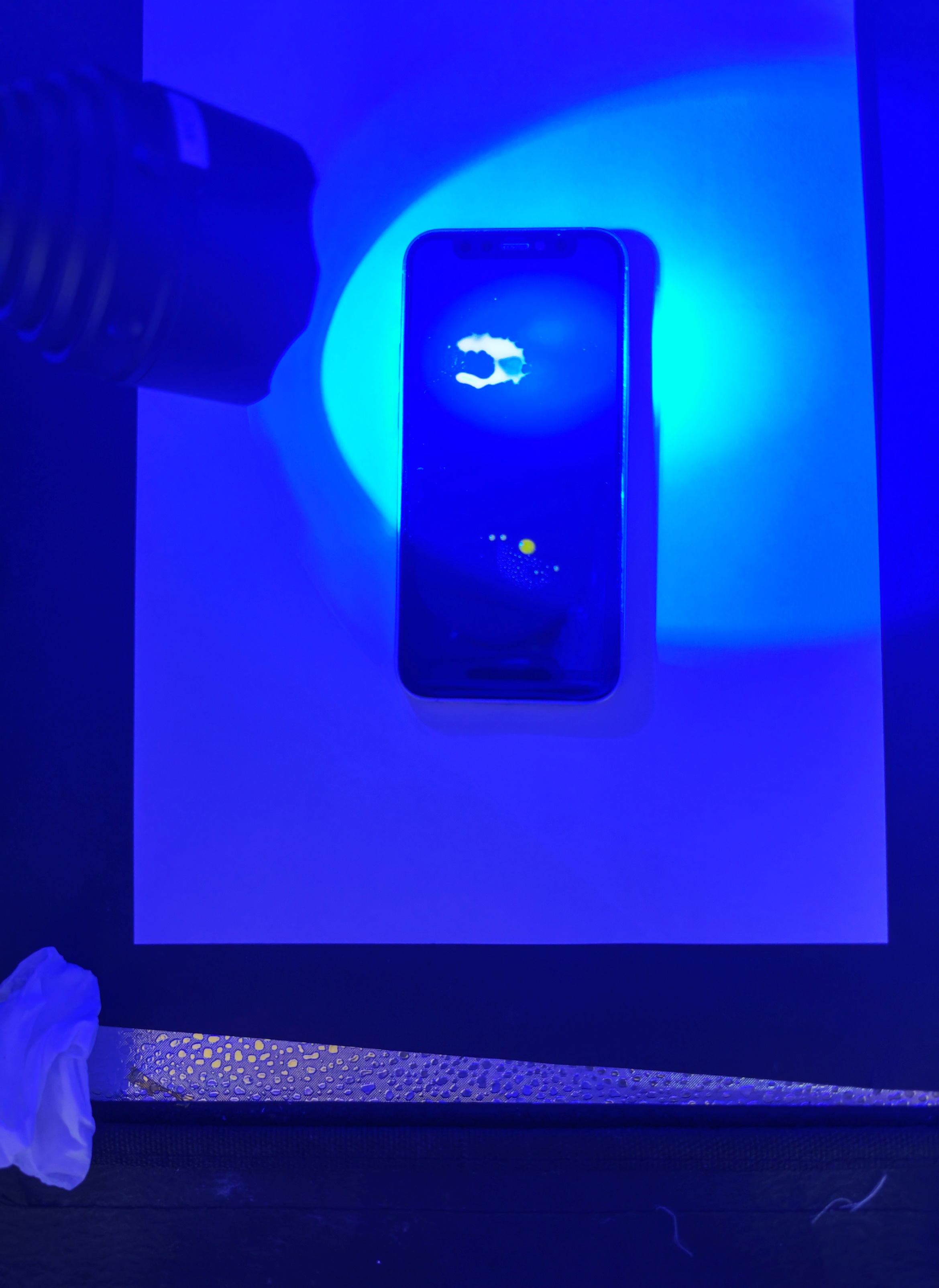


**Uncoated**

**Coated**

**Figure S11.** The difference in adhesion of the uncoated glass surface and the coated glass surface to artificial sebum solution under ultraviolet conditions.


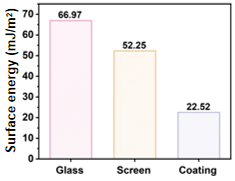


**Figure S12.** The surface energy value of the coating, screen and the glass.


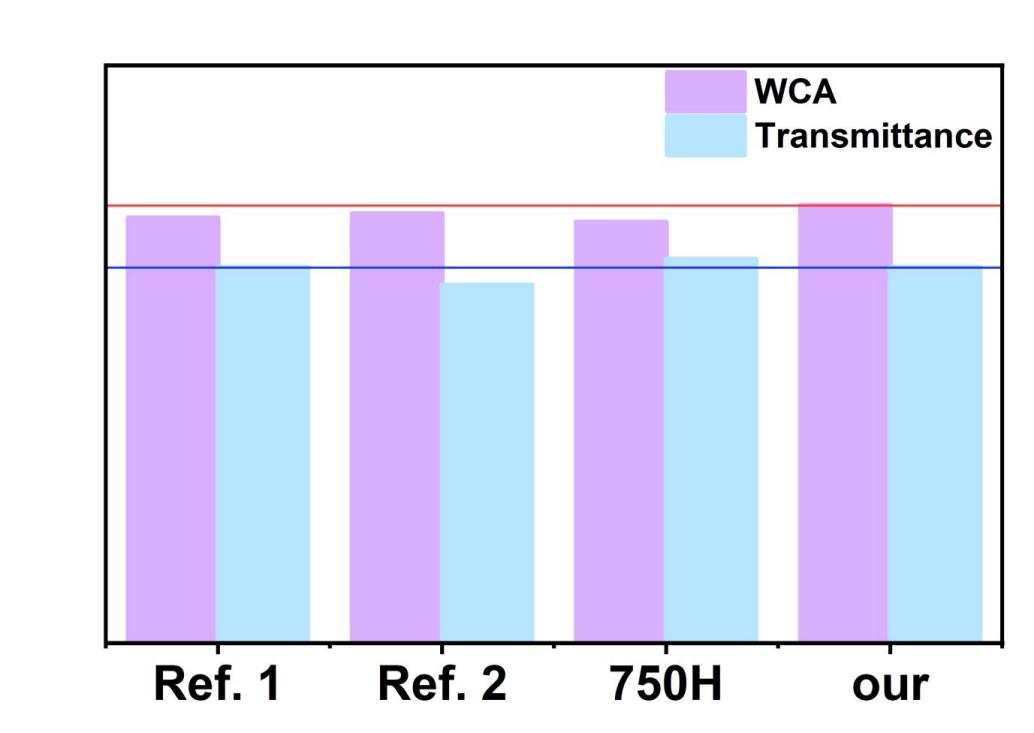


**Figure S13.** Comparison of Coating and Other Anti-fouling Products in Terms of Water Contact Angle and Transmittance.


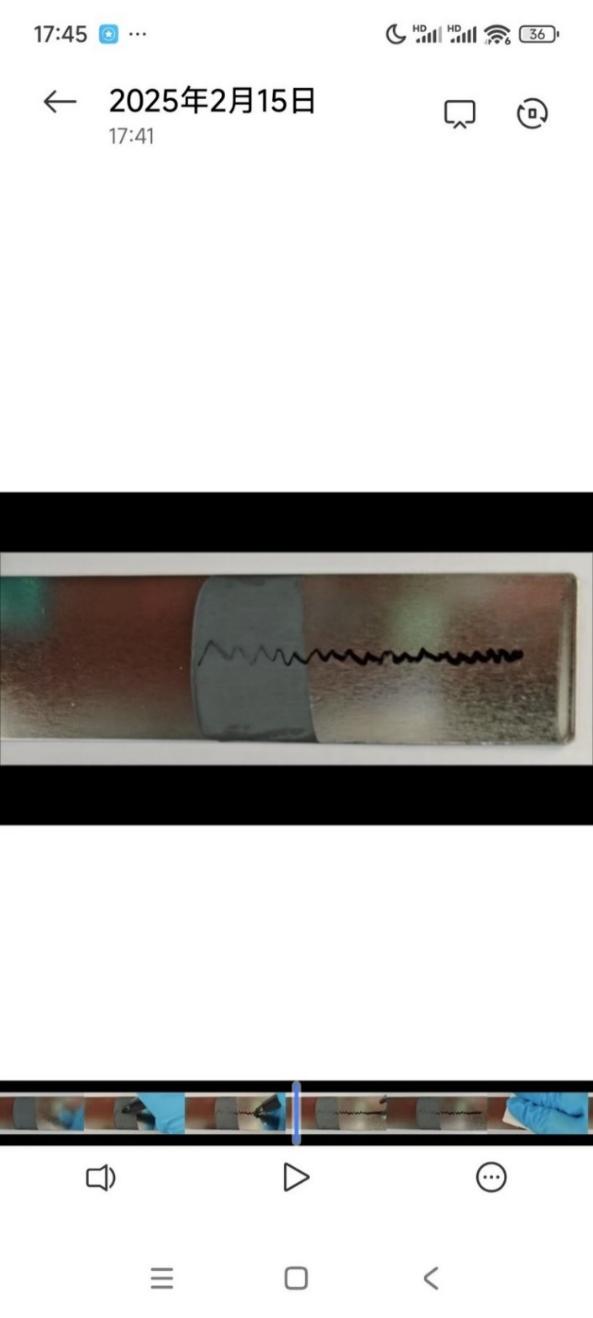

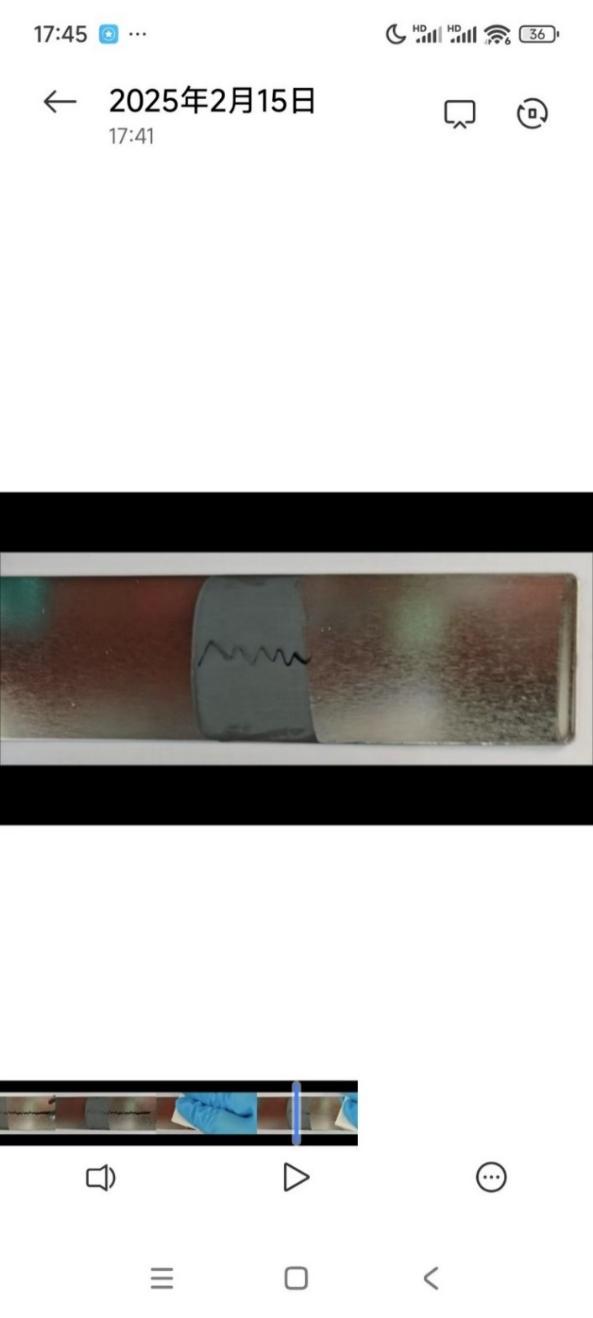


**pH = 1, 24 h**


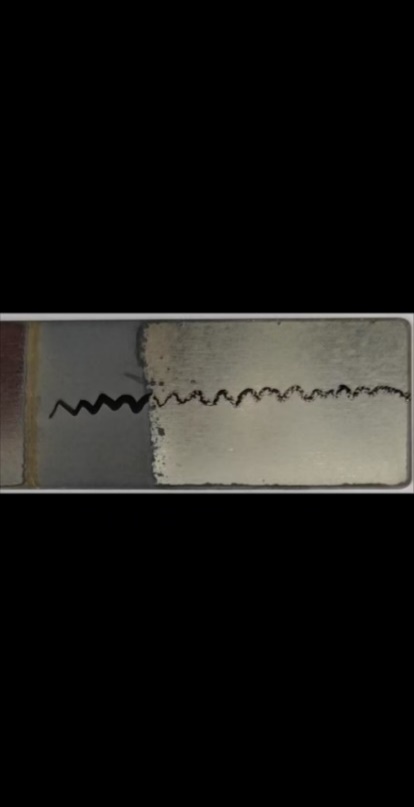

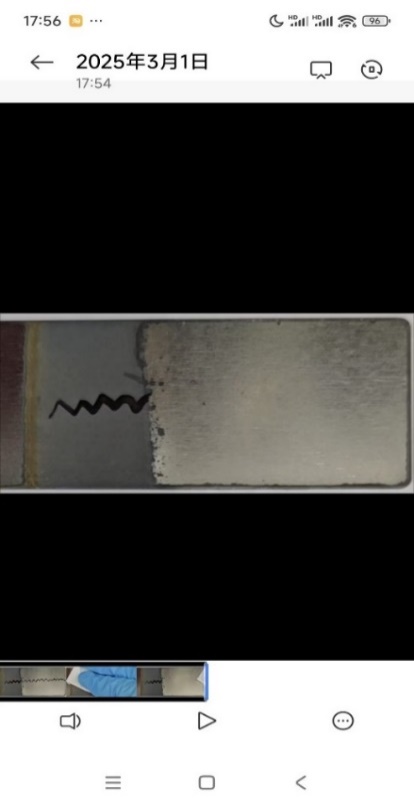


**pH = 14, 10 h**


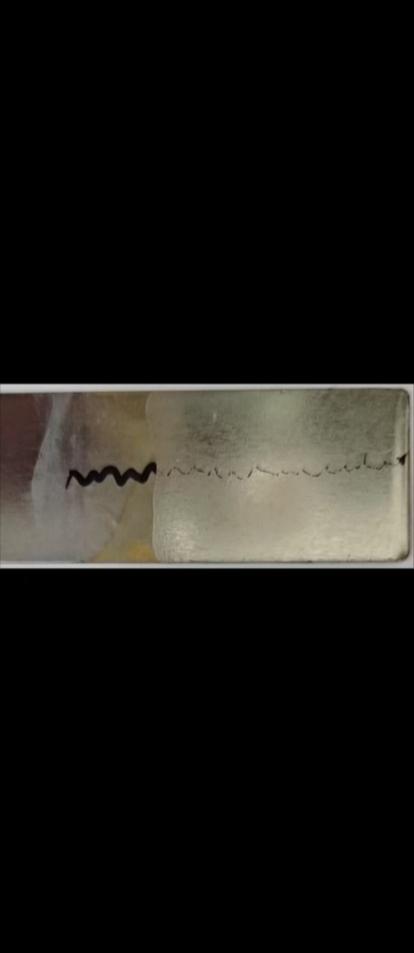

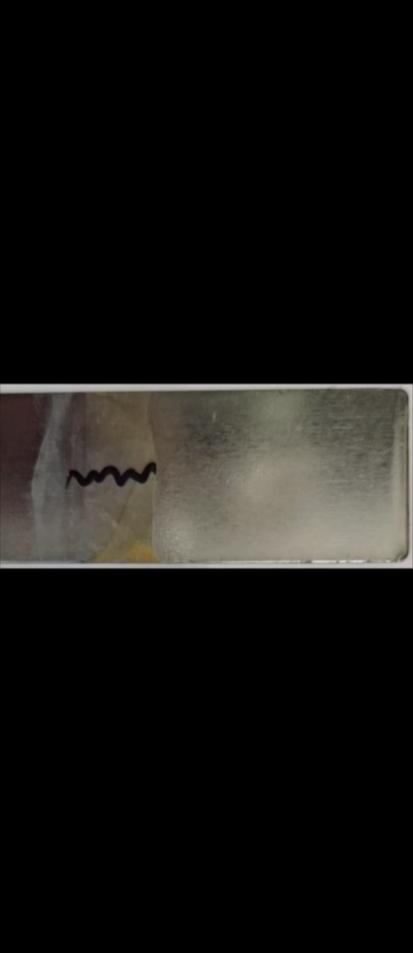


**3.5 wt% NaCl, 20 d**

**Figure S14.** Photos of the coated surface after immersion tests in strong acid, strong base and salt solutions.


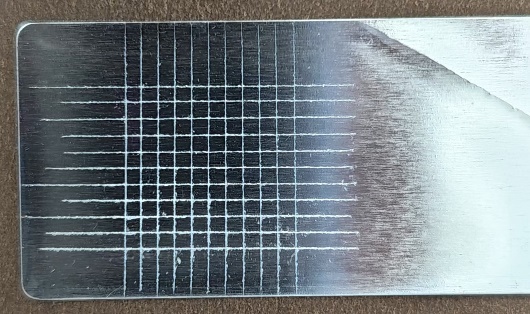

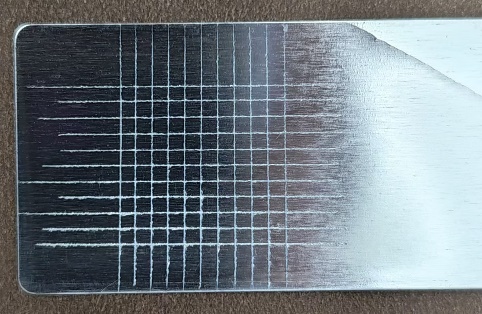

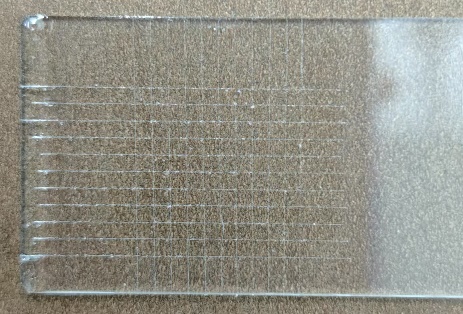

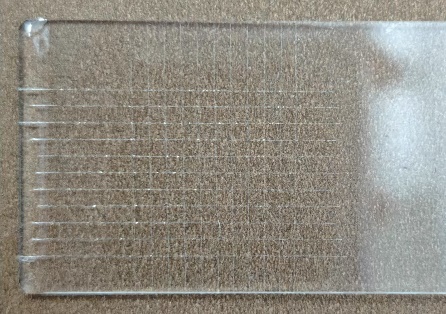

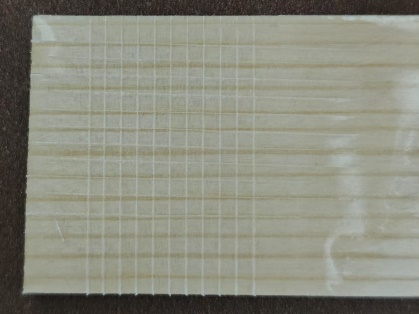

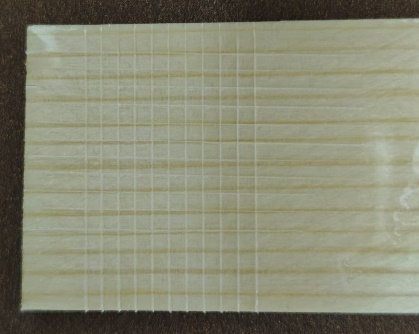

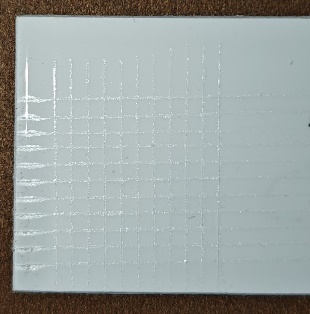

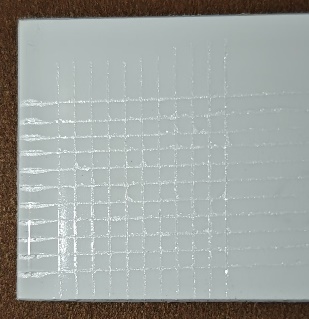


**Tinplate**

**Glass**

**Wood**

**PP**

**Figure S15.** Apply the coating onto tinplate, glass, wood boards and PP and other substrates. After complete curing, use the grid test method to evaluate the adhesion of the coating to the substrates.


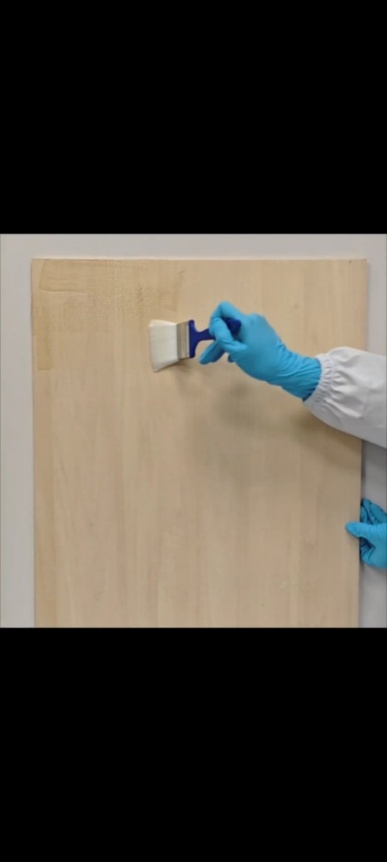

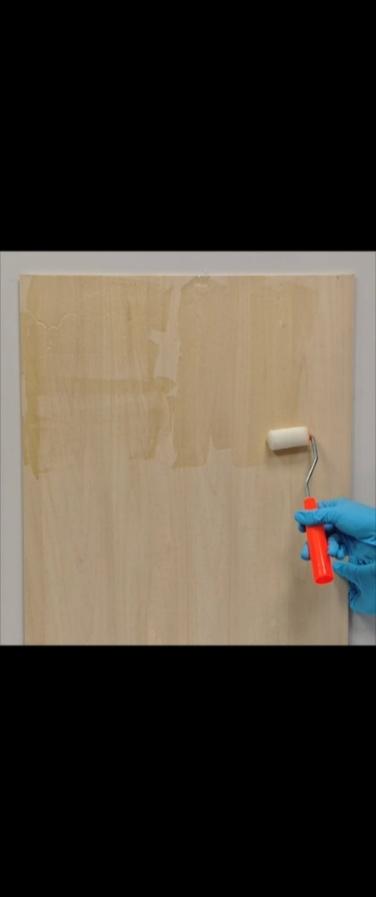


**10 cm**

**10 cm**

**Brush coating**

**Roll coating**

**Figure S16.** This resin coating can be uniformly and densely applied over a large area through industrial brushing and rolling processes.

| **Table S1: Horizontal and vertical elemental distribution** | | | | |
| --- | --- | --- | --- | --- |
| Element content（wt%） | C | N | O | Si |
| Surface | 36.48 | 2.84 | 23.82 | 36.86 |
| Cross-section | 32.51 | 2 | 30.08 | 35.41 |

| **Table S2: Atomic composition at surface and bottom surfaces** | | | | | |
| --- | --- | --- | --- | --- | --- |
| Atomic content（%） | C1s | N1s | O1s | Si2s | Si2p |
| Surface | 43.03 | 1.19 | 19.47 | 18.26 | 18.05 |
| bottom | 48.82 | 0.58 | 18.58 | 16.43 | 15.58 |

**Movie S1.** Various liquids sliding off the coated glasses.

**Movie S2.** Various coated substrates exhibited handwriting erasable performance.

**Movie S3.** The coating exhibited anti-tape adhesion.

**Movie S4.** The coating exhibited liquid repellent performance on two piece of pressed glasses.

**Movie S5.** Test of anti-sticking property of coating when immersed in cooking oil.

**Reference**

(1) Sethi, S. K.; Soni, L.; Shankar, U.; Chauhan, R. P.; Manik, G. A molecular dynamics simulation study to investigate poly(vinyl acetate)-poly(dimethyl siloxane) based easy-clean coating: An insight into the surface behavior and substrate interaction. *J. Mol. Struct.* **2020**, *1202*, 127342. DOI: https://doi.org/10.1016/j.molstruc.2019.127342.

(2) YUAN Guoming, WU Kun*, YANG Hui, LIU Zhijun, ZHANG Yuemiao, SHI Jun, YANG Li. Preparation and Properties of Hydrophobic Wear-resistant UV-curable Polysiloxane Coating. Guangzhou Chem., 2024, 49 (03), 47-51. DOI: https://doi.org/10.16560/j.cnki.gzhx.20240307.
